# Effects of a comprehensive reservation service for non-emergency registration on appointment registration rate, patient waiting time, patient satisfaction and outpatient volume in a tertiary hospital in China

**DOI:** 10.1186/s12913-019-4652-6

**Published:** 2019-11-01

**Authors:** Wanhua Xie, Xiufeng Yang, Xiaojun Cao, Peiying Liu

**Affiliations:** 1 Department of outpatient, Guangzhou Women and Children’s Medical Center, Guangzhou Medical University, Guangzhou, China; 2 Department of Science, Education and data Management, Guangzhou Women and Children’s Medical Center, Guangzhou Medical University, Guangzhou, China; 3 Department of pediatrics, Guangzhou Women and Children’s Medical Center, Guangzhou Medical University, Guangzhou, China

**Keywords:** Comprehensive reservation service for non-emergency registration, Appointment registration rate, Waiting time, Patient satisfaction, Outpatient volume

## Abstract

**Background:**

In China, a long waiting time for registration is a common occurrence in many tertiary hospitals. This study aimed to analyze the effects of a comprehensive reservation service for non-emergency registration on appointment registration rate, patient waiting time, patient satisfaction and outpatient volume at the Guangzhou Women and Children’s Medical Center.

**Methods:**

This was a cross-sectional study. This study investigated the effects of a comprehensive reservation service for non-emergency registration in Guangzhou Women and Children’s Medical Center in China starting in October 2015. In total, 2194 patients completed a satisfaction survey administered by the Guangdong Situation Research Center. The content of the questionnaire consisted of six aspects: general impression, service attitude, service quality, hospital environment, price perception and medical ethics. A Likert 5-point rating scale was used in the questionnaire; answers were classified as “very satisfied”, “relatively satisfied”, “neutral”, “unsatisfied” and “very unsatisfied”. The method of application was paper-based. T-tests were used to compare the sample means, and chi-square tests were used to compare the rates. A multiple-test procedure was performed to evaluate the differences in the reservation rates during a 12-month period.

**Results:**

After the implementation of the comprehensive reservation service for non-emergency registration in our hospital, which has an annual outpatient volume of approximately 4 million, the monthly appointment registration rate increased from (34.95 ± 2.91)% to(89.13 ± 3.12)%,*P* < 0.01. The patient waiting time was significantly reduced (P < 0.01), and the proportion of patients who believed that the waiting time required improvement was decreased significantly (*P* < 0.01). Moreover, the third-party evaluation result of outpatient satisfaction significantly improved (P < 0.01). The total hospital outpatient volume decreased(P < 0.01). The outpatient volume of the Department of General Pediatrics decreased.

**Conclusion:**

The implementation of the comprehensive reservation service for non-emergency registration in the hospital shortened patient waiting time and improved patient satisfaction, and the outpatient volume was effectively controlled. These results indicated that this program obtained the desired results in a Grade 3A hospital in China.

## Background

With the expectation of an increasing global population and an increase in overall life expectancy over the next 30 years, governments all over the world are striving to expand the scope of health services [1, 2]. In addition, waiting time in outpatient clinics are regarded as substantial obstacles and needs to be reduced worldwide [3, 4]. Clinical practice has suggested that the appointment registration process is the responsibility of hospital management. Effective appointment registration services through telephone calls and messages has an impact on appointment adherence and clinical outcomes for patients with diabetes [5]. The current outpatient service system is faced with challenges regarding the effective arrangement of appointments and providing medical services to patients [6]. Ortiz Barrios [7] suggested that high-risk pregnant women may experience serious health complications due to prolonged appointment waiting time. In China, due to the development of the family doctor system and community hospital services, the family doctor appointment system remains under development. In addition to receiving referred patients, tertiary hospitals in China receive a large number of patients with mild diseases for the first time.

Hospitals in China do not demand that patients have a prescheduled appointment, and patients can either make an appointment in advance or come directly to the hospital for registration without making a prior appointment. Many patients prefer to see a well-known expert in a large hospital. A long waiting time for registration is a common occurrence of many tertiary hospitals in China; thus, effective management and diversion have become one of the main goals of health care reform in China. The Action Plan for the Further Improvement of Medical Services, issued by the National Health and Family Planning Commission, recommends improving the appointment registration rate of tertiary hospitals in China [8].

According to the satisfaction survey conducted by the Guangdong Situation Research Center (a third party) in the first quarter of 2015, the outpatient satisfaction score of our hospital was 81.07 points. Moreover, 67.33% of outpatient respondents suggested that the first step was to improve the waiting time, which remained a key point for improving the satisfaction with outpatient services in our hospital.

To shorten the waiting time of patients, gradually promote graded diagnoses and treatments, and encourage patients to seek medical treatment in a reasonable and orderly manner, the Guangzhou Women and Children’s Medical Center implemented a comprehensive reservation service for non-emergency registration in China starting in October 2015; our hospital was the first to implement such a system in China.

The purpose of this study was to investigate the effects of a comprehensive reservation service for non-emergency registration and the optimization of medical service processes on appointment registration rate, patient waiting time, patient satisfaction regarding waiting time, satisfaction degree and outpatient volume.

## Methods

Guangzhou Women and Children’s Medical Center has implemented the comprehensive reservation service of non-emergency registration since October 2015. With the exception of emergency and isolated patients in the clinic, the remaining patients are encouraged to make an appointment using any of the following methods: a mobile phone (WeChat public platform, Alipay of Guangzhou Women and Children’s Medical Center), the Yichengtong application (mobile application), the hospital website, interclinic appointment registration or appointment registration for subsequent visits after discharge, phone calls and an on-site self-service terminal.

**Fig. 1:**
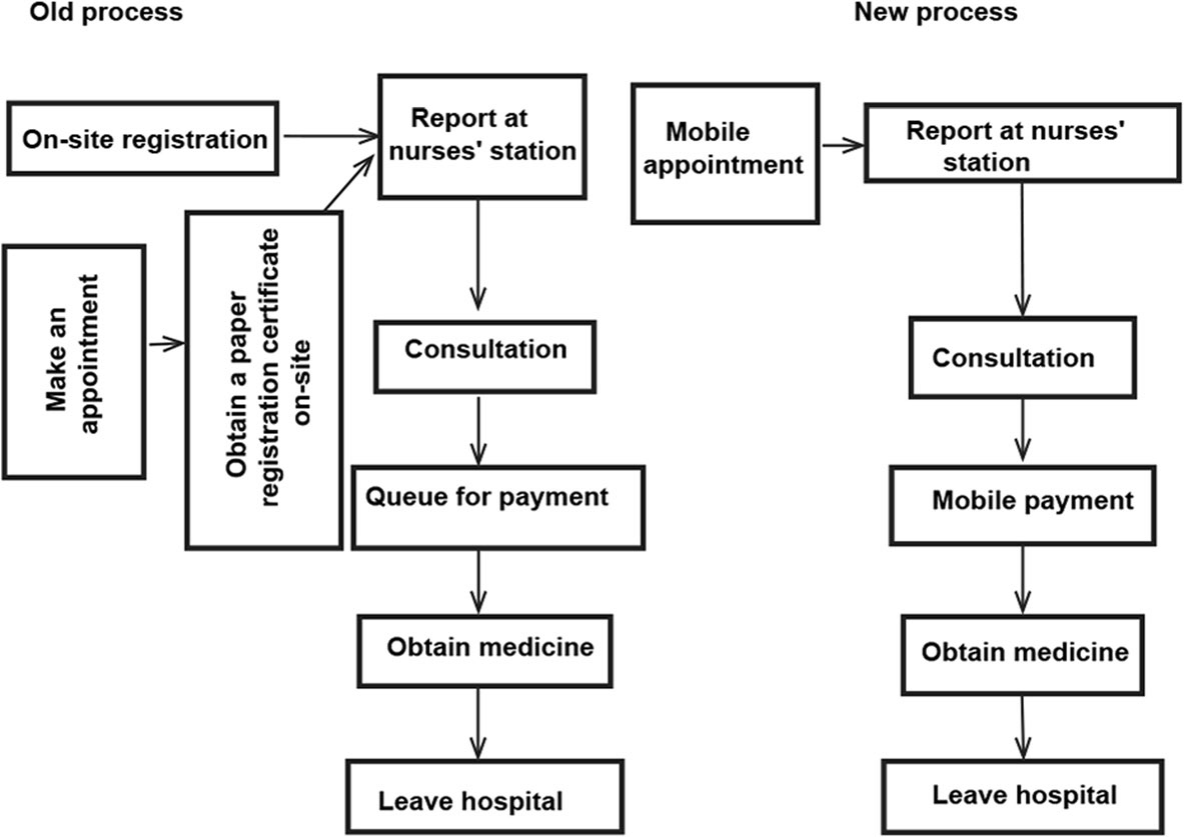
Comparison of outpatient medical service processes before and after the implementation of the comprehensive reservation service of non-emergency registration

Patients are mainly encouraged to use the mobile payment systems for online registration, as there is no need to queue for payment and obtaining a paper registration certificate in our hospital. The large number of patients who make appointments but do not pay in advance results in a large number of patients who visit the hospital to pay for a paper registration certificate after making the appointments. Among three districts with more than 12,000 outpatients per day, there was a longer queue. The comparison of outpatient medical service processes before and after the implementation of the comprehensive reservation service are shown in Fig. 1.

Combining reservation rules and information technology avoids wasting registration number resources and restricts speculative behavior. Our hospital has unified the appointment registration methods and has re-amended the appointment registration rules to strengthen the management of nonattendance at the clinic.

The Guangzhou Women and Children’s Medical Center consists of Zhujiang New Town Hospital District, Child Hospital District and Maternal and Infant Hospital District. It is the largest specialist hospital for women and children in South China, and patients from all over the country are seen.

The appointment registration rate and outpatient volume data were obtained from the Business Intelligence System of our hospital. Data on patient satisfaction and the proportion of patients who believed that the waiting time needed improvement were extracted from quarterly survey results of the outpatient satisfaction survey conducted by the Guangdong Situation Research Center. Data collection took place from October 2014 to September 2016. The satisfaction survey was conducted on-site in the women and children’s medical center. The method of application was paper-based. The survey was conducted by 60 trained investigators. During the first quarter of 2015, 2194 valid questionnaires were collected from the outpatient clinics; 982 questionnaires were collected from the outpatient clinic of Zhujiang New Town hospital District, 884 questionnaires were collected from the outpatient clinic of the Children’s Hospital District, and 328 questionnaires were collected from the outpatient clinic of the Maternal and Infant Hospital District.

General satisfaction was the first-level indicator; there were six second-level indicators: general impression, service attitude, service quality, hospital environment, price perception and medical ethics. There were 31 third-level indicators. The responses to the third-level index were categorized by a 5-point Likert scale. The answers were coded from 1 to 5: 1-very satisfied, 2-satisfied, 3-neutral, 4-unsatisfied, and 5-very unsatisfied [9, 10]. All of the questionnaire data were statistically analyzed by SPSS software. The current internationally recognized method for calculating the satisfaction degree on the basis of public opinion was adopted for calculating each of the third-level indexes [9, 10]:100 points: “very satisfied”, 80 points: “relatively satisfied”, 60 points: “neutral”, 40 points: “unsatisfied”, 20 points: “very unsatisfied”; “unclear” was assigned for missing values, which were excluded from the data analysis.The minimum and maximum possible values of the third-level indexes were 20 and 100, respectively (Additional file 1).

General satisfaction was calculated by summing the scores of all the second-level indicators after multiplying them by their assigned weights: general impression (5%), service attitude (15%), service quality (20%), hospital environment (10%), price perception (20%), and medical ethics (30%) [9, 10].

### Statistical analyses

IBM SPSS Statistics 23.0 software was used for the statistical analyses. The W test was used to evaluate whether the data were normally distributed. Independent samples t test were used to compare the sample means of registration waiting time, consultation waiting time and outpatient satisfaction. Paired t-tests were used to compare the sample means of monthly outpatient volume, and a chi-square test was used to compare the rates. The multiple test procedure was performed to evaluate the differences in the reservation rates during a 12-month period. To correct for multiple comparisons, the *p* value of each variable was adjusted to a family-wise corrected α of 0.05 using bootstrap permutation testing in SAS 9.4 for Windows software (SAS Institute, Inc., Cary, NC, USA 2015). *P* < 0.05 was considered statistically significant.

## Results

Regarding the questionnaires, female respondents accounted for 53.19%, male respondents accounted for 46.81%, Guangzhou residents accounted for 68.91%, respondents living in other cities of Guangdong Province accounted for 26.47%, respondents living in other provinces accounted for 4.39%, and others accounted for 0.23%.

The appointment registration rate was significantly increased after the implementation of the comprehensive reservation service for non-emergency registration.As shown in Table 1, our hospital had an annual outpatient volume of approximately 4 million, and the monthly appointment registration rate was significantly increased from (34.95 ± 2.91)% to (89.13 ± 3.12)%(*P* < 0.01).

**Table 1:** Comparison of the monthly appointment reservation rates before and after the implementation of the comprehensive reservation service for non-emergency registration

	Before implementation(%)	After implementation (%)	P
January	33.06	89.61	0.000
February	34.72	88.65	0.000
March	33.14	90.50	0.000
April	33.22	90.20	0.000
May	33.13	88.13	0.000
June	33.80	88.85	0.000
July	39.28	90.97	0.000
August	40.33	92.56	0.000
September	39.46	91.43	0.000
October	33.22	80.10	0.000
November	32.99	88.72	0.000
December	33.05	89.82	0.000
Mean value	34.95 ± 2.91	89.13 ± 3.12	0.000

*the *P* value was adjusted to a family-wise corrected α of 0.05 using bootstrap permutation testing

The hospital waiting time for patients and their family members were reduced. As shown in Table 2, the patient waiting time was significantly reduced after the implementation of the comprehensive reservation service (*P* = 0.000, *P* < 0.01).

**Table 2:** Comparison of the waiting time for registration and waiting time for consultation before and after the implementation of the comprehensive reservation service for non-emergency registration

Mode	Before implementation(minute)	After implementation(minute)	Time saved (minute)	t	p
Waiting time for registration	25.05 ± 8.17	1.00 ± 0.21	24.05 ± 7.31	131.999	0.000
Waiting time for consultation	60.30 ± 9.26	12.00 ± 3.00	48.30 ± 9.73	223.402	0.000

*P* < 0.05: statistically significant

As shown in Table 3, after the implementation of the comprehensive reservation service, the proportion of patients who believed that the waiting time needed improvement was significantly decreased (*P* < 0.01).

**Table 3:** Comparison of the proportion of patients who believed that waiting time needed improvement before and after the implementation of the comprehensive reservation service for non-emergency registration

Time	Before implementation(%)	Time	After implementation(%)	Difference between the latter and the former (%)	P
First quarter of 2015	59.76	First quarter of 2016	48.58	↓ 11.18	0.000
Second quarter of 2015	67.33	Second quarter of 2016	49.80	↓ 17.53	0.000
Third quarter of 2015	54.59	Third quarter of 2016	45.42	↓ 9.17	0.000
Fourth quarter of 2014	62.16	Fourth quarter of 2015	48.81	↓ 13.35	0.000

*P* < 0.05: statistically significant

As shown in Table 4, compared with 1 year before the implementation of the comprehensive reservation service, the quarterly satisfaction of outpatients was significantly increased 1 year after the implementation of the service (*P* < 0.01).

**Table 4:** Comparison of outpatient satisfaction before and after the implementation of the comprehensive reservation service for non-emergency registration

Time	Before implementation (points: mean ± SD)	After implementation (points: mean ± SD)	t	P
First quarter	80.32 ± 13.30	87.11 ± 13.28	16.529	0.000
Second quarter	82.18 ± 13.36	86.47 ± 13.47	10.348	0.000
Third quarter	80.51 ± 13.59	87.86 ± 14.32	17.053	0.000
Fourth quarter	81.68 ± 13.60	86.64 ± 14.48	11.439	0.000
Overall mean value	81.17 ± 13.90	87.02 ± 13.62	13.749	0.000

*P* < 0.05:statistically significant

**Fig. 2:**
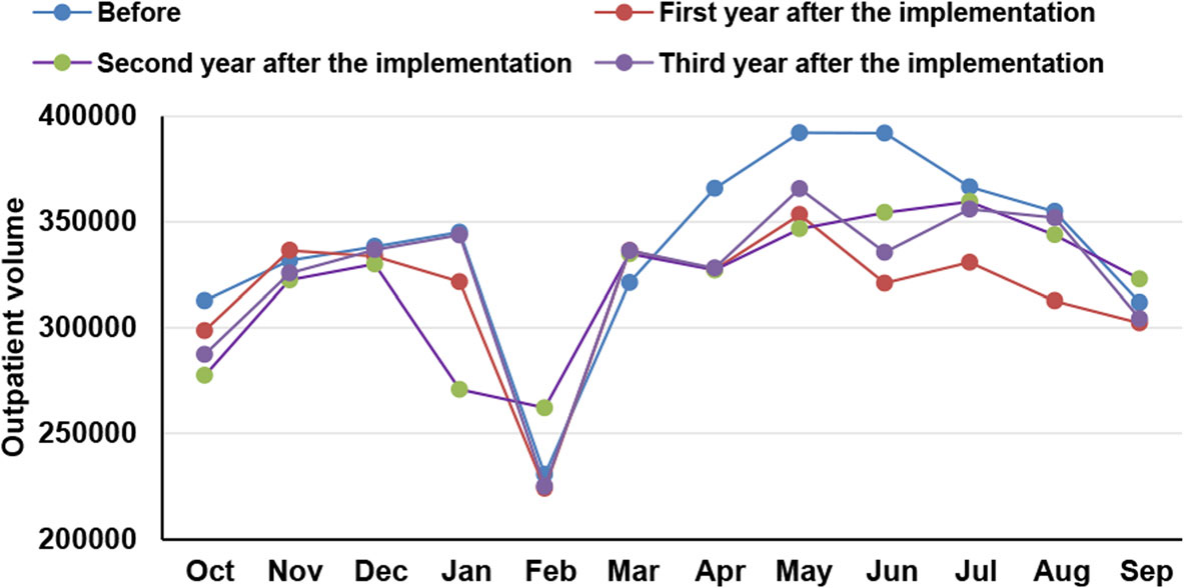
Monthly outpatient volume one year before and three years after the implementation of the comprehensive reservation service for non-emergency registration

The total hospital outpatient volume decreased, and the Department of General Pediatrics outpatient volume decreased.Compared with 1 year before the implementation of the comprehensive registration service, the monthly total outpatient volume during the first year after implementation was significantly decreased (P < 0.01) (Table 5). The comparison of monthly outpatient volumes 1 year before and after implementation is shown in Fig. 2. At 1 year after the implementation of the comprehensive registration service, the outpatient volume of the Department of General Pediatrics was 829,059, which was decreased by 11.32% compared to the year before implementation. The proportion of the outpatient volume of the Department of General Pediatrics to the total outpatient volume at 1 year after the implementation of the comprehensive registration service was 21.83%, which was decreased compared to 1 year before the implementation of the comprehensive registration service (23.01%).

**Table 5:** Comparison of the annual monthly outpatient volume before and after the implementation of the comprehensive reservation service for non-emergency registration

Year	Monthly outpatient volume	Difference	t	P
One year before implementation	338,552 ± 43,654	–	–	–
One year after implementation	316,520 ± 32,876	22,032 ± 24,153	3.160	0.009

*P* < 0.05: statistically significant

## Discussion

Our study showed that the patient waiting time was significantly decreased after implementing the comprehensive reservation service for non-emergency registration. The proportion of patients who believed that waiting time needed improvement was decreased, and outpatient satisfaction was improved significantly. The total outpatient volume was lower than that before the implementation of the comprehensive reservation service, indicating that the outpatient volume was effectively controlled. After the implementation of the comprehensive reservation service for non-emergency registration, the medical service process was simplified and optimized by mobile registration and payment. Our hospital shared our experience of implementing a comprehensive reservation service for non-emergency registration with colleagues in other hospitals throughout the country. On January 3, 2018, our hospital was awarded the “2015-2017 National Quality Service Demonstration Hospital” award.

The significant increase in the appointment registration rate was evaluated. China’s medical system is obviously defined based on Chinese characteristics. In European and North American countries [11–13], most clinicians require patients to make appointments in advance for medical consultations. If a patient has a disease but no emergent condition, the primary care provider directly contacts a specialist in a hospital and makes arrangements for further diagnosis and treatment of the patient.

The average waiting time of patients was decreased after the implementation of the comprehensive reservation service for non-emergency registration.Our experience showed that improvements in service optimization processes also reduced the difficulty in seeing a doctor, consistent with the opinions of many researchers in China and abroad [14, 15]. The registration service could facilitate improved follow-up of patients. Moreover, it is the basis of continuous service, and the need for registration service is also the consensus of medical service researchers [16]. Singapore has established percentile targets for appointment lead-times for patients because appointment postponements are regarded as unacceptable for health care services [17]. Recent studies have shown that the accessibility for patients can be significantly improved by applying simple methods and performing barrier analyses [18].

This study was different from those in other countries, which might be because of the following reasons. The development of a variety of patient-friendly appointment registration methods was necessary to successfully implement the comprehensive reservation service for non-emergency registration. A previous survey study showed that the dimensional score of “promoting appointment services and effectively diverting patients” was decreased [19]. The study found that whether the appointment registration rate could be improved or not depended on the convenience of the appointment registration method. The previous study agreed with our results. Mobile applications collect data from patients regarding their eating habits, body weight and overall health status through weekly surveys [20]. As our hospital is the largest women and children’s hospital in South China, patients come to our hospital from all over the country. Some of the patients come from large cities, while some of the patients come from rural areas. Moreover, the education levels of patients are different; there are young patients as well as some elderly patients with children. Considering these factors, our hospital offered a several methods for registration as previously mentioned; patients chose their method of registration according to their preference.

Because of the mobile payment systems for online registration, there was no need to obtain a paper registration certificate at the hospital, which shortened the waiting time. A long waiting time for a medical consultation has become an important factor affecting outpatient satisfaction [21, 22]. It was necessary to conduct more research on medical health applications in children’s health care centers to ensure the quality and reliability of mobile health applications [23].

Activities to strengthen publicity should be conducted through media campaigns. Pidgen TE [24] noted that parents had limited information regarding appointment setting. Therefore, we solicited the opinions and suggestions of patients and their family members as well as clinical departments to develop the “Explanation of 17 common problems of patients and their family members” and “Implementation details of the comprehensive reservation service for non-emergency registration” guidance documents. To ensure understanding, these documents contain plain language and cartoons and other pictures. In addition, our hospital has carried out many promotional campaigns employing various media platforms such as television, websites, the WeChat public platform, newspapers, display screens, leaflets and so on.

This study showed that the total outpatient volume of our hospital was decreased and effectively controlled after the implementation of the comprehensive reservation service for non-emergency registration, further assisting in grading diagnosis and treatment, consistent with the results of Wang who noted that the outpatient volume in a department was decreased after health care reform in Beijing [25]. Before the implementation of the comprehensive reservation service for non-emergency registration, the hospital admitted all patients who visited the hospital. After the implementation of the comprehensive reservation service, medical resources were allocated in an organized way. For example, when the appointment of the Department of General Pediatrics was full, patients with some common complaints such as cold and fever could visit the nearest district-level hospital, and those who needed actual specialist treatment could be seen at our hospital. This, in turn, decreased the outpatient volume of the Department of General Pediatrics and decreased the total outpatient volume. The role of tertiary hospitals was thus played, and the grading diagnosis and treatment were also observed. Referral service is an important part of medical reform in China [26]. Continuous attention should be paid to the reservation and referral tracking [27].

### Limitations

The results of this study are based on the research data of one hospital, the Guangzhou Women and Children’s Medical Center. Additionally, the data collection time was limited.

## Conclusion

In this study, the changes after the implementation of the comprehensive reservation service for non-emergency registration in a Chinese hospital were summarized. To optimize medical service processes and meet the needs of patients, hospitals need to improve processes and information distribution. The implementation of the comprehensive reservation service for non-emergency registration decreased the patient waiting time and improved patient satisfaction, indicating that this program achieved the intended results in a Grade 3A Hospital in China.

## Supplementary information

**Additional files 1.** Attachment1-Satisfaction Questionnaire.

## Data Availability

The datasets used and/or analyzed in the current study are available from the corresponding author upon reasonable request.
