# Supplementary material for: Effects of a comprehensive reservation service for non-emergency registration on appointment registration rate, patient waiting time, patient satisfaction and outpatient volume in a tertiary hospital in China
Source: BMC Health Serv Res. 2019 Nov 1;19:782. doi: 10.1186/s12913-019-4652-6 (PMC6824126; doi:10.1186/s12913-019-4652-6)
Supplement: Supplementary file 1 — Additional files 1. Attachment1-Satisfaction Questionnaire. [file 12913_2019_4652_MOESM1_ESM.doc]

**Service Satisfaction Questionnaire, Guangzhou Women and Children’s Medical Center (Outpatient Questionnaire)**

Dear Madam/Sir:

To improve the quality of medical services in Guangzhou Women and Children’s Medical Center and improve your experience in diagnosis and treatment, we are entrusted by the Guangzhou Women and Children Medical Center to conduct this service satisfaction survey. Please fill out the questionnaire according to the actual situation and actual experience during your visit. Unless otherwise indicated, there is only one correct answer for each question. Content that relates to your personal privacy will be kept confidential in accordance with the “Statistics Act”. Thank you for your support and participation! I wish you a speedy recovery.

If you have any questions, please contact us: (020) 66315101.

Guangdong Situation Research Center

**October 2015**

**Screening questions**

**※A. Completed by** 1. Patient 2. Relative or friend

**※B.** **Sex of the patient 1**. Male 2. Female

**※C. Age of the patient** 1. Under 1 year 2.1-6 years 3.7-14 years 4.15-18 years 5.19-25 years 6. 26-35 years 7.36-45 years 8.46–55 years 9.56-65 years 10. Above 66 years

**Questionnaire**

1. **In which department was your outpatient visit？**

(1) Branch __________________________ (2) Department:__________________

2.**How do you feel about the hospital overall? (Please select one answer)**

(1) Very satisfied (2) Quite satisfied (3) Neutral (4) Unsatisfied (5) Very unsatisfied (6)Unclear

**3. Please indicated your satisfaction with service attitude**

| **Evaluation**  **Content** | Very satisfied | Quite satisfied | Neutral | Unsatisfied | Very unsatisfied | Unclear |
| --- | --- | --- | --- | --- | --- | --- |
| (1) Medical guides |  |  |  |  |  |  |
| (2) Doctors |  |  |  |  |  |  |
| (3) Nurses |  |  |  |  |  |  |
| (4) Radiology staff (X-ray, MRI, CT, etc.) |  |  |  |  |  |  |
| (5) Laboratory staff (finger prick, urine, stool vaginal discharge, etc.) |  |  |  |  |  |  |
| (6)Functional department staff (B-ultrasound, EEG, ECG, etc.) |  |  |  |  |  |  |
| (7) Pharmacy staff |  |  |  |  |  |  |
| (8) Interns/refreshers |  |  |  |  |  |  |

4.**Service quality**

**(1) Your acceptable queue time for payment/medication dispensing/waiting is (please select one answer):**

1) Within 10 minutes 2) 10-15 minutes 3) 15-20 minutes

4) 20-30 minutes 5) 30-60 minutes 6) Longer than 60 minutes

**(2) In the description of the service quality, please place a “√” in the box that matches your actual experience. Please select “Not experienced” for medical services you did not obtain:**

| **Evaluation**  **Content** | Very satisfied | Quite satisfied | Neutral | Unsatisfied | Very unsatisfied | Not experienced |
| --- | --- | --- | --- | --- | --- | --- |
| 1) Appointment and registration |  |  |  |  |  |  |
| 2) Level of diagnosis and treatment of the doctors |  |  |  |  |  |  |
| 3)Triage nurses and assisting doctors |  |  |  |  |  |  |
| 4)Therapeutic outcome |  |  |  |  |  |  |
| 5) Window service queue time for payment/ medication dispensing |  |  |  |  |  |  |
| 6)Waiting time |  |  |  |  |  |  |
| 7) Doctors’ enquiry and communication |  |  |  |  |  |  |
| 8) Information from the doctor regarding expected expenses |  |  |  |  |  |  |
| 9) Rehabilitation treatment |  |  |  |  |  |  |
| 10) Decoction |  |  |  |  |  |  |

**(3) How do you feel about the service provided by the intern/refresher?**

**1) Was the intern/refresher guided?**

a. Yes b. No c. Not applicable (Please skip to Section 5, medical environment)

**2) Please indicate your satisfaction with the service provided by intern/refresher：**

| **Evaluation**  **Content** | Very satisfied | Quite satisfied | Neutral | Unsatisfied | Very unsatisfied | Not experienced |
| --- | --- | --- | --- | --- | --- | --- |
| a. Service attitude of the intern/refresher |  |  |  |  |  |  |
| b. Nursing skills, such as blood drawing and injection, of the intern/refresher |  |  |  |  |  |  |
| c. Diagnosis and treatment by intern/refresher doctor |  |  |  |  |  |  |
| d. Operational skills of the intern/refresher technicians |  |  |  |  |  |  |
| e. Medication guidance by the intern/refresher pharmacist |  |  |  |  |  |  |

**5. Please indicate your satisfaction with the medical environment**

| **Evaluation**  **Content** | Very satisfied | Quite satisfied | Neutral | Unsatisfied | Very unsatisfied | Unclear |
| --- | --- | --- | --- | --- | --- | --- |
| (1) The cleanliness of the treatment room and medical equipment |  |  |  |  |  |  |
| (2) The clarity of indications for outpatient treatment |  |  |  |  |  |  |
| (3)The comfort level of the waiting room seats |  |  |  |  |  |  |

**6. Please indicate your satisfaction with the medical cost**

| **Evaluation**  **Content** | Very satisfied | Quite satisfied | Neutral | Unsatisfied | Very unsatisfied | Unclear |
| --- | --- | --- | --- | --- | --- | --- |
| (1)Drug cost |  |  |  |  |  |  |
| (2) Examination cost |  |  |  |  |  |  |

**7. Medical ethics and style**

**(1) Have you ever given the doctor gift money when you visited the hospital? (please select one answer)**

1) Yes, it was not returned 2).Yes, but the doctor returned it to me 3) No 4) Unclear

**(2) Have you encountered any of the following conditions during your medical treatment? (multiple answers can be provided)**

1) The doctor prescribed expensive drugs or more drugs than required (please explain ____________________________)

2)I underwent unnecessary examinations (please explain ____________________________________)

3) The doctor prevaricated or refused to provide treatment (please explain ________________________________)

4) The hospital medical staff asked for gift money (explicitly or implicitly) (please explain _____________________)

5)No experience with the above situations

**8. Loyalty and advice**

**(1) Loyalty**

**1) Will you choose this hospital if you need medical service again? (please select only one answer)**

a. Yes b. No c. Not sure

**2) Are you willing to recommend our hospital to your relatives and friends (please select only one answer)**

a. Very willing b. Willing c. Neutral d. Unwilling e. Very unwilling

**(2) Others**

**1) What is the main reason you chose this hospital? (multiple answers can be selected)**

a. Convenient time b. Trusted medical techniques c. Good service attitude d. Convenient transportation e. Convenient procedure f. Good medical environment g. Good medical equipment h. Reasonable cost i. Referral from other hospitals or recommended by relatives or friends j. Designated hospital by the insurance agency k.Media promotion l. Other

**2) Aspects that urgently need improvement (multiple answers can be selected)**

a. Order control b. Medical environment c. Equipment d. Service attitude e. Privacy protection f. Waiting time g. Cost h. Diagnosis and treatment level i. Medical ethics and style j. Appointment and registration k. Doctor-patient communication l. Indicators m. Others

**(3)Your most pertinent comments or suggestions about the hospital：**

**Basic information of the respondent (the following information is confidential and is for statistical purposes only)**

**Payment method:** 1. Self-funded 2. Insurance 3. Paid by company 4. Two or all payment methods were involved

**Residence:** 1. Local resident 2. Other cities in Guangdong Province

3. Another province 4. Other

**Education level:** 1. Elementary school or below 2. Junior high school 3. Senior high school or secondary specialized school 4. Diploma 5. Bachelor’s degree 6. Master’s degree or above

**Occupation:** 1. Worker or casual worker, temporary worker, hourly worker 2. Farmer 3. General employee 4. Manager 5. Social organization staff 6. Civil servant or institution staff 7. Self-employed or private business owner 8. Retired 9. Unemployed 10. Freelancer 11. Other

**Name of respondent： Contact number:**

**Note: Your phone number will only be used to review the authenticity of the questionnaire once at most.**
